# Fatal Fruit: Unveiling the Risks and Responses to Ackee Poisoning in a South Florida Inpatient Unit

**DOI:** 10.7759/cureus.117006

**Published:** 2026-09-27

**Authors:** Shalondria J Sears, Enrique Gondar, Kevin Sowden, Jessene Aquino-Thomas, Ruchir Chokshi

**Affiliations:** 1 College of Medicine, Florida Atlantic University Charles E. Schmidt College of Medicine, Boca Raton, USA; 2 Internal Medicine, Broward Health Medical Center, Fort Lauderdale, USA; 3 Invasive Plant Research Lab, US Department of Agriculture (USDA) Agricultural Research Lab, Fort Lauderdale, USA

**Keywords:** ackee poison, acute metabolic encephalopathy, foodborne toxins, hypoglycin, ingestion of toxic materials, interdisciplinary care, jamaica, jamaican vomiting sickness, sudden early death, toxic plant

## Abstract

The ackee fruit is a staple in many Afro-Caribbean diets. Yet, when consumed in its unripe form, it can induce Jamaican vomiting sickness, a potentially fatal condition caused by hypoglycin, a toxin that disrupts fatty acid oxidation and gluconeogenesis. Although cases are well-documented in endemic regions, reports outside the Caribbean remain rare. We report the case of a 33-year-old male with no comorbidities who presented in South Florida with acute metabolic encephalopathy, severe hypoglycemia, and profound electrolyte disturbances following the ingestion of unripe ackee fruit. Despite early recognition and aggressive interventions, including glucose administration, supportive therapy, and management coordinated by internal medicine, neurology, nephrology, toxicology, intensive care, and palliative care teams, the patient’s clinical condition continued to deteriorate over 21 days post-exposure. This case is distinctive for its occurrence in a non-endemic region, the absence of comorbidities, and the refractory course despite multidisciplinary intervention. It highlights both the diagnostic and therapeutic challenges of ackee poisoning in unfamiliar clinical settings. Increased globalization and the availability of culturally specific foods in diverse regions may increase the likelihood of similar presentations outside endemic areas. Awareness among healthcare providers is therefore essential. Ackee poisoning should be considered in patients presenting with unexplained hypoglycemia and encephalopathy, particularly in communities with Afro-Caribbean dietary practices. Early recognition, timely intervention, and interdisciplinary collaboration remain critical to improving patient outcomes. This case underscores the importance of provider awareness regarding culturally specific dietary exposures and their potential for severe toxicological consequences.

## Introduction

*Blighia sapida* (ackee) is a tropical fruit native to West Africa and widely consumed in Caribbean communities [1-4]. Jamaica’s national fruit is traditionally eaten with savory dishes for breakfast. The edible portion consists of yellow arils atop black seeds, enclosed in a pod that splits upon ripening. Fully ripe arils are safe, but unripe arils, seeds, raphe, and rind contain high levels of hypoglycin A and B, with hypoglycin A responsible for the most toxicity [5,6]. In the United States, only canned or processed ackee from FDA-approved growers is available [7].

Ingestion of unripe ackee can cause Jamaican vomiting sickness (JVS), a potentially fatal condition characterized by nonketotic hypoglycemia, metabolic encephalopathy, and electrolyte disturbances. Cases peak during winter months when unripe fruit is more likely to be consumed, highlighting the link between seasonal epidemiology and toxin exposure. Hypoglycin A is metabolized to methylene cyclopropyl acetic acid (MCPA), which inhibits β-oxidation by binding acyl CoA dehydrogenases and carnitine-related enzymes, leading to systemic toxicity [8,9]. Neurological manifestations, including seizures, which are observed in most fatal cases, altered mental status, paresthesia, and tremors, reflect disease severity [10]. Recognition of recent ackee ingestion and dose-dependent toxicity is critical, as recovery typically occurs within one week with supportive care.

## Case presentation

We present the case of a previously healthy, 33-year-old male with no known past medical history. Family history was noncontributory. Social history was notable for recent travel to the Caribbean; no known substance use, occupational exposures, or high-risk behaviors were reported.

Approximately 12 hours after ingesting ackee and saltfish for dinner and again the following morning, the patient developed persistent, cyclical vomiting. Ackee fruit ingestion is known to cause Jamaican vomiting sickness due to hypoglycin A toxicity, which disrupts fatty acid metabolism and leads to severe hypoglycemia [6,11,12]. Symptoms progressed over the next 48 hours, with worsening dehydration and confusion. On day 3 from symptom onset, he experienced a witnessed seizure, prompting presentation to a local emergency department.

At initial presentation, the patient was found to have severe metabolic encephalopathy with a Glasgow Coma Scale score of 6 (eye opening to pain, incomprehensible verbal response, withdrawal from pain). Laboratory evaluation revealed severe hypoglycemia (serum glucose 3.5 mmol/L), anemia (hemoglobin 4.3 g/dL), and mild hypoxia (oxygen saturation 92%). Computed tomography of the brain demonstrated hypodensity in the basal ganglia, concerning for hypoxic-ischemic encephalopathy [2,10].

Initial management included intravenous dextrose (dextrose 50) and fluid resuscitation, resulting in transient improvement in mentation and glucose levels. However, recurrent hypoglycemia and intermittent seizures persisted, requiring treatment with midazolam and phenytoin.

During the first week of hospitalization (days 3-10 from symptom onset), the patient developed progressive bone marrow suppression with worsening anemia. Severe systemic toxicity from ackee ingestion has been associated with multiorgan dysfunction [7,13]. Due to religious objections to blood transfusion, he was treated with Eprex. Despite temporary stabilization, his clinical status deteriorated, necessitating intubation for airway protection.

His course was further complicated by enterococcal bacteremia, multiorgan failure, and a small iatrogenic pneumothorax requiring chest tube placement. Antibiotic therapy included vancomycin and levofloxacin.

On day 14 from initial symptom onset, due to persistent altered mental status and lack of clinical improvement, the patient was transferred to Broward Regional Hospital for higher-level care.

Upon arrival, the patient was sedated and mechanically ventilated with a chest tube and Foley catheter in place. He was afebrile and hemodynamically stable (blood pressure 116/72 mmHg) with adequate oxygenation on ventilatory support. Laboratory studies demonstrated leukocytosis, severe macrocytic anemia, transaminitis with impaired hepatic synthetic function, electrolyte abnormalities, and acute kidney injury consistent with prerenal azotemia (Table 1).

**Table 1:** Pertinent lab results on admission to the emergency department AST: aspartate aminotransferase, ALT: alanine aminotransferase, PT: prothrombin time, PTT: partial thromboplastin time, INR: international normalized ratio

Complete Metabolic Panel		Lab Reference Ranges
Sodium	170 mmol/L	136-146 mmol/L
Potassium	2.8 mmol/L	3.5-5.0mmol/L
Bicarbonate	21 mmol/L	22-28 mmol/L
Chloride	140 mmol/L	95-105 mmol/L
Blood Urea Nitrogen	43 mg/dL	7-18 mg/dL
Creatinine	1.7 mg/dL	0.6-1.2 mg/dL
Glomerular Filtration Rate	51 mL/min/1.73 m²	>90mL/min/1.73 m²
Calcium	7.0 mg/dL	8.4-10.2 mg/dL
Albumin	2.0 g/dL	3.5-5.5 g/dL
Total Protein	5.7 g/dL	6.0-7.8 g/dL
AST	86 U/L	12-38 U/L
ALT	228 U/L	10-40 U/L
Total Creatine Kinase	1104 U/L	25-90 U/L (males)
Brain Natriuretic Peptide	184 pg/mL	<100 pg/mL
Total Bilirubin	0.9 g/dL	0.1-1.0 mg/dL
Complete Blood Count With Differential		
White Blood Cell Count	17.16 x 10³/uL	4.5-11.0 x 10^15^uL
Red Blood Cell Count	1.24 x 10⁶/uL	20-36 x 10^3^/uL
Hemoglobin	4.3 g/dL	13.5-17.5 g/dL
Hematocrit	14%	41-53%
Mean Corpuscular Volume	112.9 fL	80-100 fL
Red Cell Distribution Width	13%	11.5-14.5%
Platelet count	294 x 10^3^ /uL	150 - 400 x 10^16^ /uL
Coagulation Profile		
PT	16.9 s	11-15 s
INR	1.4	0.8-1.2
PTT	29.1 s	25-40 s

Urinalysis revealed pyuria, hematuria, and numerous budding yeast. Urine culture subsequently grew *Candida tropicalis* (Table 2).

**Table 2:** Urinalysis on admission WBC: white blood cell; RBC: red blood cell

Component	Result	Reference Range
Color	Yellow	Yellow
Appearance	Turbid	Clear
Glucose	Normal	Absent
Bilirubin	Negative	Absent
Ketones	Negative	Absent
Blood	Large Abnormal	Absent
pH	6.0	5.0-7.5
Leukocyte Esterase	Small Abnormal	Absent
Nitrite	Negative	Absent
WBC	62/hpf	0-5/hpf
RBC	574/hpf	0-2/hpf
Bacteria	Rare Abnormal	Absent/Few
Budding Yeast	Many Abnormal	Absent
Reflex to Urine Culture	Yes	

Neuroimaging with non-contrast CT of the brain showed no acute intracranial abnormalities (Figure 1).

**Figure 1:**
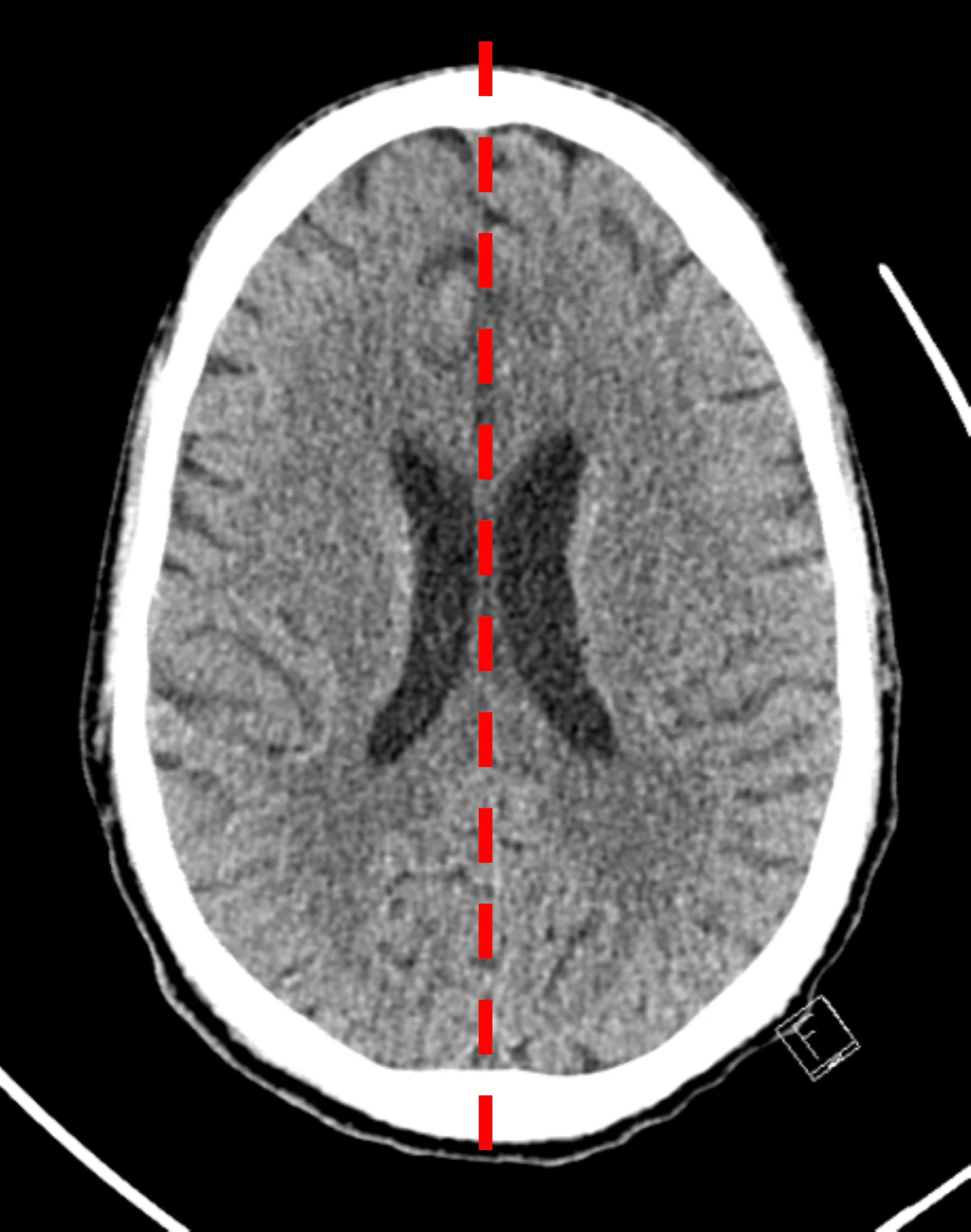
Axial non-contrast computerized tomography scan of the brain The image shows preserved gray-white matter differentiation and symmetrical ventricles (red dashed line). No midline shift or visible acute intracranial abnormalities, suggesting further classification of the patient’s altered mental status.

Computed tomography of the abdomen and pelvis demonstrated free fluid in the pelvis of unclear etiology (Figure 2).

**Figure 2:**
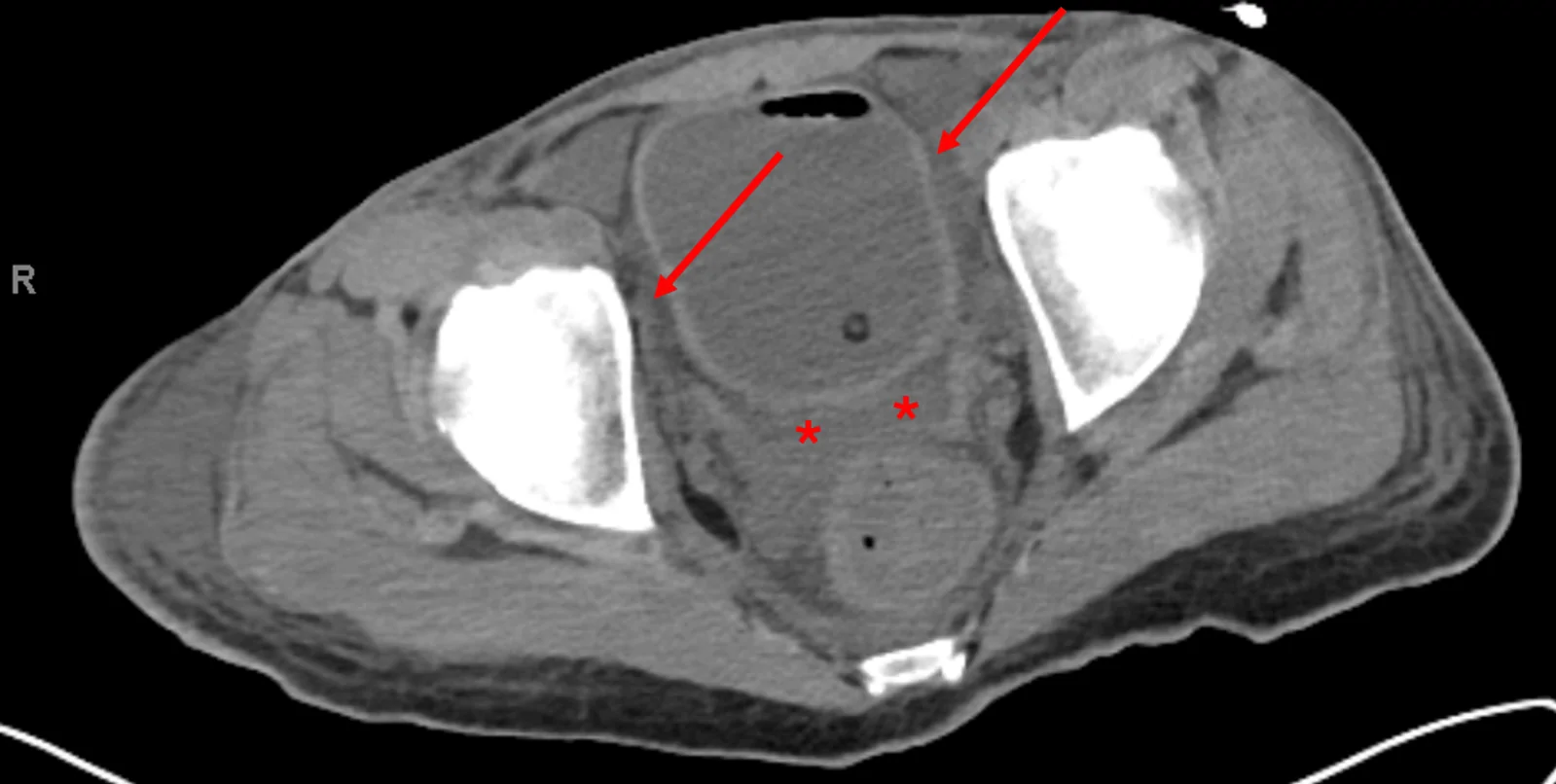
Non-contrast axial computed tomography scan of the abdomen and pelvis Free fluid of unknown origin in the rectovesicular pouch (red asterisks). Additionally, fluid collections are visible bilaterally in the anterior pelvis adjacent to the bladder (red arrows). These findings suggest the presence of intra-abdominal fluid accumulation, possibly indirectly due to hypoglycin effects on the liver and kidneys, leading to secondary complications.

Computed tomography of the chest revealed bilateral airspace disease with ground-glass opacities and a small left apical pneumothorax (Figure 3).

**Figure 3:**
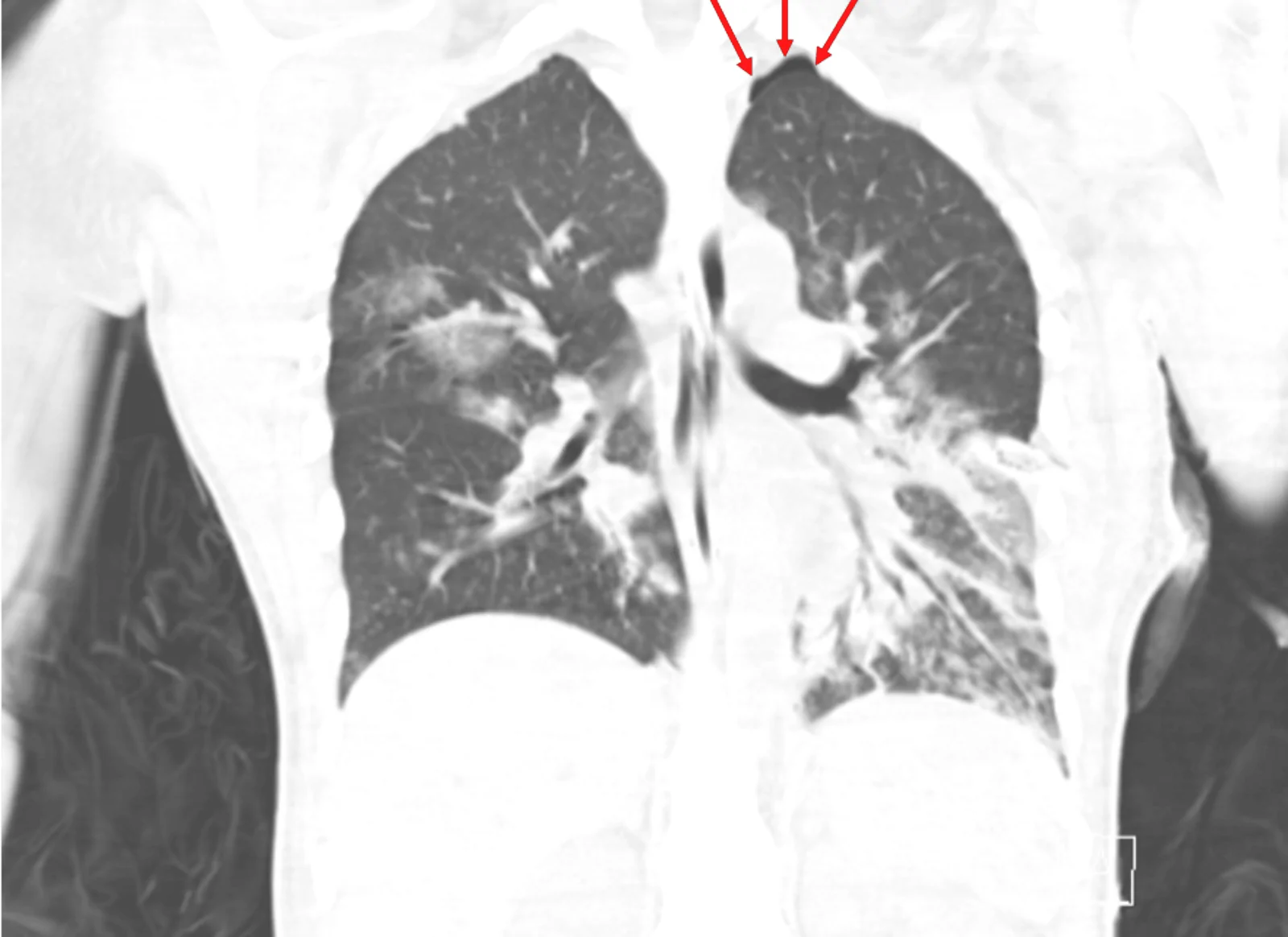
Non-contrast computed tomography scan of the chest Extensive bilateral airspace disease, as demonstrated by patchy ground-glass opacities visible in the upper lobes with a lower lobe predominance, is demonstrated. A left-sided chest tube is in place, and a small apical left-sided pneumothorax is indicated by red arrows.

Chest radiograph demonstrated left-sided infiltrates consistent with pneumonia (Figure 4).

**Figure 4:**
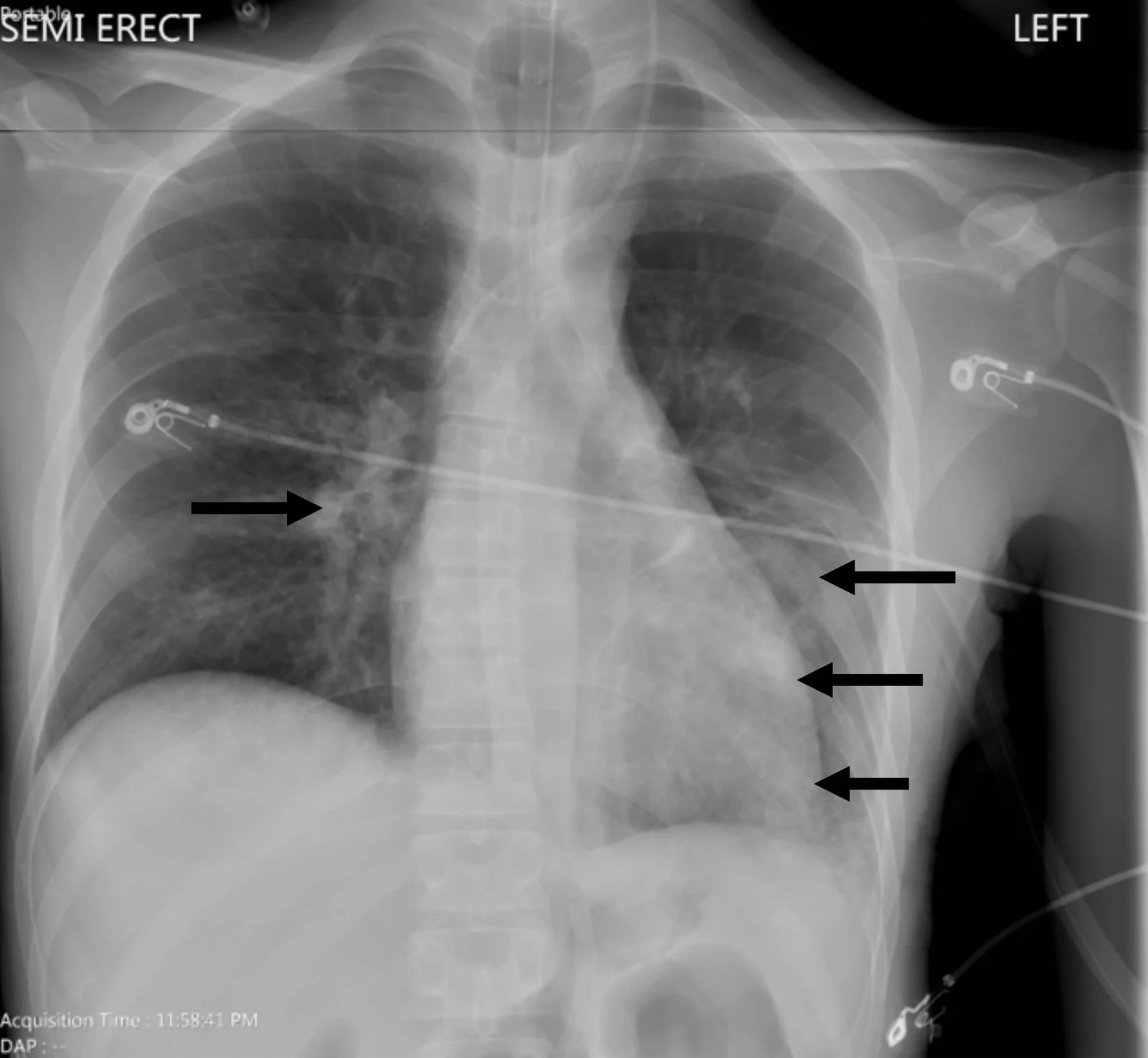
X-ray of the chest The image shows a chest X-ray with notable ground-glass opacities, predominantly visible in the left lung base and hilar region. Black arrows highlight these areas of increased opacity, which appear as hazy, grayish areas against the normally dark lung fields. This radiographic finding is significant and may indicate various underlying pathologies, requiring further clinical correlation.

Tracheal aspirate cultures grew *Acinetobacter baumannii*, prompting escalation of antibiotics to include Meropenem, later de-escalated to Zosyn.

Magnetic resonance imaging of the brain performed on hospital day 2 (day 16 from symptom onset) demonstrated diffuse cortical diffusion restriction and T2 hyperintensities consistent with hypoxic-ischemic encephalopathy (Figure 5).

**Figure 5:**
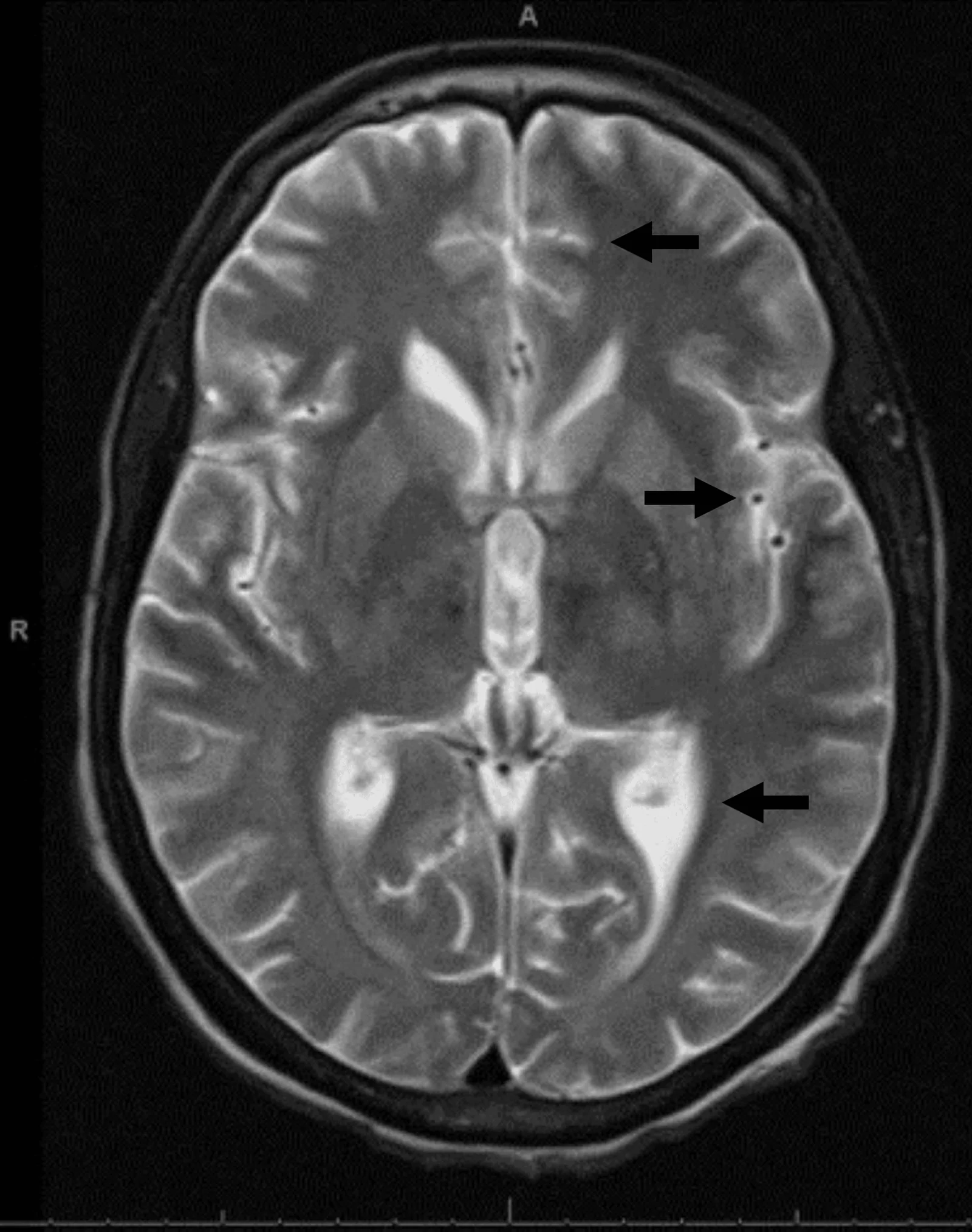
T2-weighted magnetic resonance imaging of the brain T2-weighted magnetic resonance imaging of the brain demonstrating diffuse hyperintense signals throughout the cerebral white matter. Black arrows show hyperintensities appearing as bright areas against the darker background, indicating increased water content or potential pathological changes in the affected regions.

Despite aggressive management, the patient’s condition continued to decline. He developed progressive multiorgan failure and worsening cytopenias. Treatment included dextrose 5 in water for hypernatremia and hematologic support with Aranesp, Ferrlecit, and Darbepoetin alfa.

By hospital day 7 at our institution (day 21 from symptom onset), the patient’s prognosis remained poor, and palliative care was consulted. The patient subsequently expired due to complications of severe hypoglycemic encephalopathy and multiorgan failure secondary to ackee fruit toxicity.

Written informed consent for publication was obtained from the patient’s legally authorized representative.

## Discussion

Ackee fruit, when fully ripened and properly prepared, is generally considered safe and is widely consumed in Afro-Caribbean diets [11,12]. However, ingestion of unripe or improperly prepared ackee can result in Jamaican vomiting sickness due to hypoglycin A toxicity. As shown in Figure 6, the edible portion of the fruit is the aril, which contains the toxin when immature.

**Figure 6:**
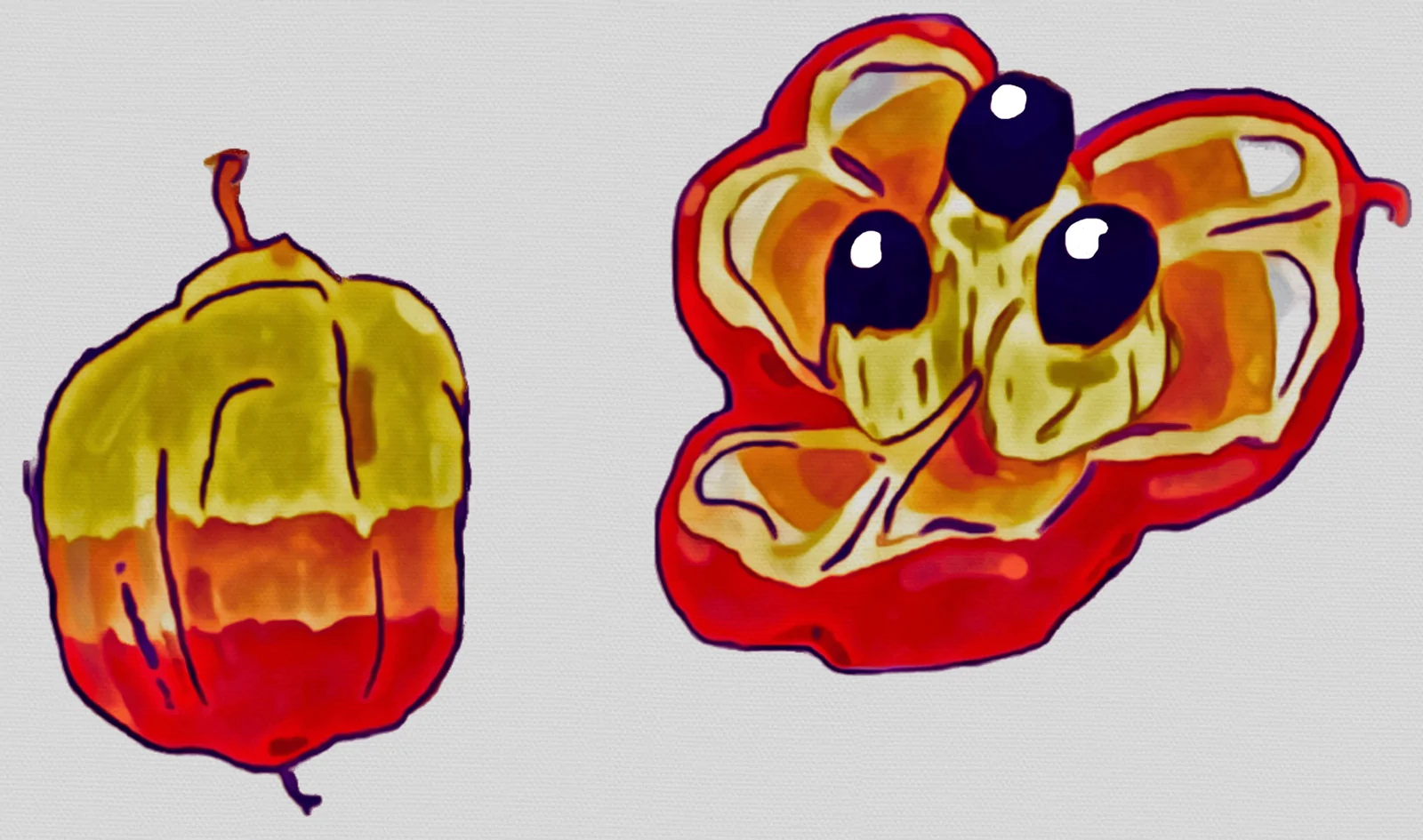
Ripe ackee fruit Ripe ackee fruit showing exposed arils and seeds. Original artwork by J. Aquino; included for educational purposes to aid recognition of the fruit associated with toxicity.

Hypoglycin A disrupts fatty acid metabolism by inhibiting beta-oxidation and gluconeogenesis, leading to profound hypoglycemia and depletion of glycogen stores [3,5,6,13]. This metabolic derangement explains the clinical presentation of vomiting, altered mental status, seizures, and, in severe cases, coma or death 12. In this case, the patient’s early symptoms of persistent vomiting followed by severe hypoglycemia and neurologic decline are consistent with this known pathophysiology. The subsequent development of hypoxic-ischemic encephalopathy highlights the consequence of prolonged untreated or refractory hypoglycemia.

This case is notable for several reasons. First, it demonstrates a severe and ultimately fatal presentation of ackee toxicity in a previously healthy adult, whereas much of the literature emphasizes pediatric or outbreak-related cases [2,10]. Second, the delayed presentation-approximately 48 hours after ingestion-likely contributed to disease severity and progression to multi-organ failure. Third, the persistence of hypoglycemia despite treatment and the extent of neurologic injury underscore the potential for irreversible complications even with aggressive management.

Prior reports have described severe outcomes, including hepatic failure and encephalopathy, following ackee ingestion [10], as well as outbreaks linked to unripe fruit consumption [2]. However, fatal cases in otherwise healthy adults remain relatively uncommon, further emphasizing the clinical significance of this presentation.

Clinically, this case reinforces several important diagnostic and management principles. Early recognition of toxin-mediated hypoglycemia is critical, particularly in patients presenting with cyclical vomiting and neurologic symptoms following travel or dietary exposure. A detailed dietary history is essential, as identification of ackee ingestion can rapidly guide diagnosis. Prompt and sustained glucose repletion remains the cornerstone of treatment, though, as demonstrated in this case, may not prevent progression in delayed or severe presentations.

Limitations of this report include incomplete access to initial outside hospital records, limiting precise characterization of early management and timing of interventions. Additionally, confirmatory toxicologic testing for hypoglycin A was not available, and the diagnosis was made clinically based on history and presentation. As a single case report, generalizability is limited.

The primary clinical takeaway from this case is the importance of early identification of ackee toxicity in patients with unexplained hypoglycemia and vomiting, particularly in the setting of recent travel or Caribbean food exposure. Recognition of this pattern may allow for earlier intervention and improved outcomes.

## Conclusions

Ackee fruit toxicity remains an important and potentially fatal cause of hypoglycemic encephalopathy. This case highlights that even previously healthy adults are at risk for severe disease, particularly with delayed presentation. Early recognition of the characteristic triad of vomiting, hypoglycemia, and altered mental status is essential for timely management.

Clinicians should maintain a high index of suspicion in patients with relevant dietary exposure, as prompt diagnosis and treatment may reduce morbidity and mortality. Increased awareness among both healthcare providers and the public is critical to preventing delayed presentations and severe complications.
